# MAFLD mediates the association between CHR and gallstones in the U.S. adults: evidence from NHANES 2021–2023

**DOI:** 10.1186/s12876-025-03805-2

**Published:** 2025-04-17

**Authors:** Xin Feng, Xiangyu Song, Xi’an Yang, Fuxiang Luan, Yufei Gu, Fengyu Zheng, Huahu Guo, Shishi Qiao

**Affiliations:** 1 Department of Hepatobiliary Surgery, The First Affiliated Hospital of Zhengzhou University, Zhengzhou, Henan 450052 China; 2 Department of General Surgery, The First Affiliated Hospital of Henan University, Kaifeng, Henan 475001 China; 3 Department of Comprehensive Surgery, Shaanxi Provincial People’s Hospital, Xi’an, Shaanxi 710068 China

**Keywords:** Gallstones, Metabolic associated fatty liver disease, High-sensitivity C-reactive protein, High-density lipoprotein cholesterol, NAHNES, Cross-sectional studies

## Abstract

**Background:**

Gallstones, a global hepatobiliary disorder, are linked to systemic inflammation, lipid disturbances, and metabolic-associated fatty liver disease (MAFLD). This population-based study aims to investigate the association of the novel inflammation-lipid composite biomarker high-sensitivity C-reactive protein-to-HDL cholesterol ratio (CHR) with gallstones and evaluate whether MAFLD mediates this relationship.

**Methods:**

This cross-sectional analysis utilized data from the National Health and Nutrition Examination Survey (NHANES, 2021–2023) to assess the correlation between the CHR and gallstone prevalence through weighted logistic regression. To evaluate potential nonlinear relationships and assess heterogeneity across key demographics, restricted cubic splines (RCS) were employed to model the association, complemented by subgroup analyses stratified by age, sex, and other covariates. A mediation analysis was used for elucidating the mediating effects of MAFLD.

**Results:**

Among 4,078 participants, 432 (10.60%) had gallstones. After adjusting for confounders, each unit increase in CHR was associated with a 165% increased risk of gallstones (OR: 2.65, 95% CI: 1.43–4.93, *P* = 0.006). The RCS curve demonstrated a nonlinear association between the CHR and gallstones (*P*_overall_ < 0.001, *P*_nonlinear_ < 0.001). Mediation analysis indicated that MAFLD explained 27.1% of this association.

**Conclusions:**

CHR is positively associated with gallstones, with MAFLD partially mediating this relationship. Managing CHR levels and preventing MAFLD may reduce gallstone incidence.

## Background

Gallstones are a prevalent hepatobiliary disease worldwide that cause significant economic and social burdens [1, 2]. The incidence rate of gallstone disease in Europe and America is approximately 10–15% [3, 4]. In 2018, the United States spent approximately $20 billion on gallstone treatments [5]. Acute cholecystitis and pancreatitis are often caused by the obstruction of the biliary tract or inflammation caused by gallstones, especially acute pancreatitis, which has a poor prognosis [6–9]. Therefore, identifying appropriate clinical indicators to alleviate the occurrence of gallstones and manage patients with gallstones is very important.

Previous studies have suggested that lipid metabolism disorders and inflammation may contribute to gallstone formation [10, 11]. In a study of the Kailuan cohort, high-sensitivity C-reactive protein (hs-CRP) was identified as an independent causal indicator of first-onset gallstones [12]. Chen et al. found a causal influence of blood lipids on gallstone formation, and lower high-density lipoprotein cholesterol (HDL-C) levels were associated with gallstone occurrence [13]. The hs-CRP-to-HDL-C ratio (CHR) is a novel composite indicator that combines markers of inflammation and lipid metabolism. Its clinical relevance is underscored by the pivotal role of chronic inflammation and dyslipidemia in the pathogenesis of gallstone formation [14]. Gao et al. demonstrated that the CHR has the potential to predict cardiovascular disease in elderly adults [15]. However, no study has clarified the association between the CHR and gallstones.

Metabolic-associated fatty liver disease (MAFLD), a common chronic liver disease, is caused by metabolic disorders [16]. Numerous recent reports have discussed the association between MAFLD and gallstones. In their review, Slouha et al. reported that patients with MAFLD had an increased risk of developing gallstones [17]. A Mendelian randomization study suggested that MAFLD is a potential causative factor of gallstones [11]. However, the detailed mechanisms and whether the link between CHR and gallstones is mediated by MAFLD have not been elucidated.

In this investigation, we hypothesized that the CHR is related to an increased risk of developing gallstones and that MAFLD plays a partial mediating role in this association. This hypothesis is based on evidence suggesting that inflammation and dyslipidemia are key contributors to both MAFLD and gallstone formation [18, 19]. The National Health and Nutrition Examination Survey (NHANES) is designed to assess the health and nutritional status of adults and children in the United States. The objective of this research was testing this hypothesis using the NHANES dataset and employing advanced statistical models to evaluate the effects of the CHR on gallstones and the mediating influence of MAFLD. The validation of this hypothesis could enhance our understanding of the potential pathophysiological mechanisms within metabolic dysregulation and gallstone disease, potentially leading to improved diagnostic and therapeutic strategies for patients with gallstones.

## Methods

### Study design and population

This cross-sectional study obtained two-year clinical data from the NHAENES database from August 2021 to August 2023. The National Center for Health Statistics (NCHS) authorized NHANES ethics approval, and all individuals signed a detailed written consent [20].

**Fig. 1:**
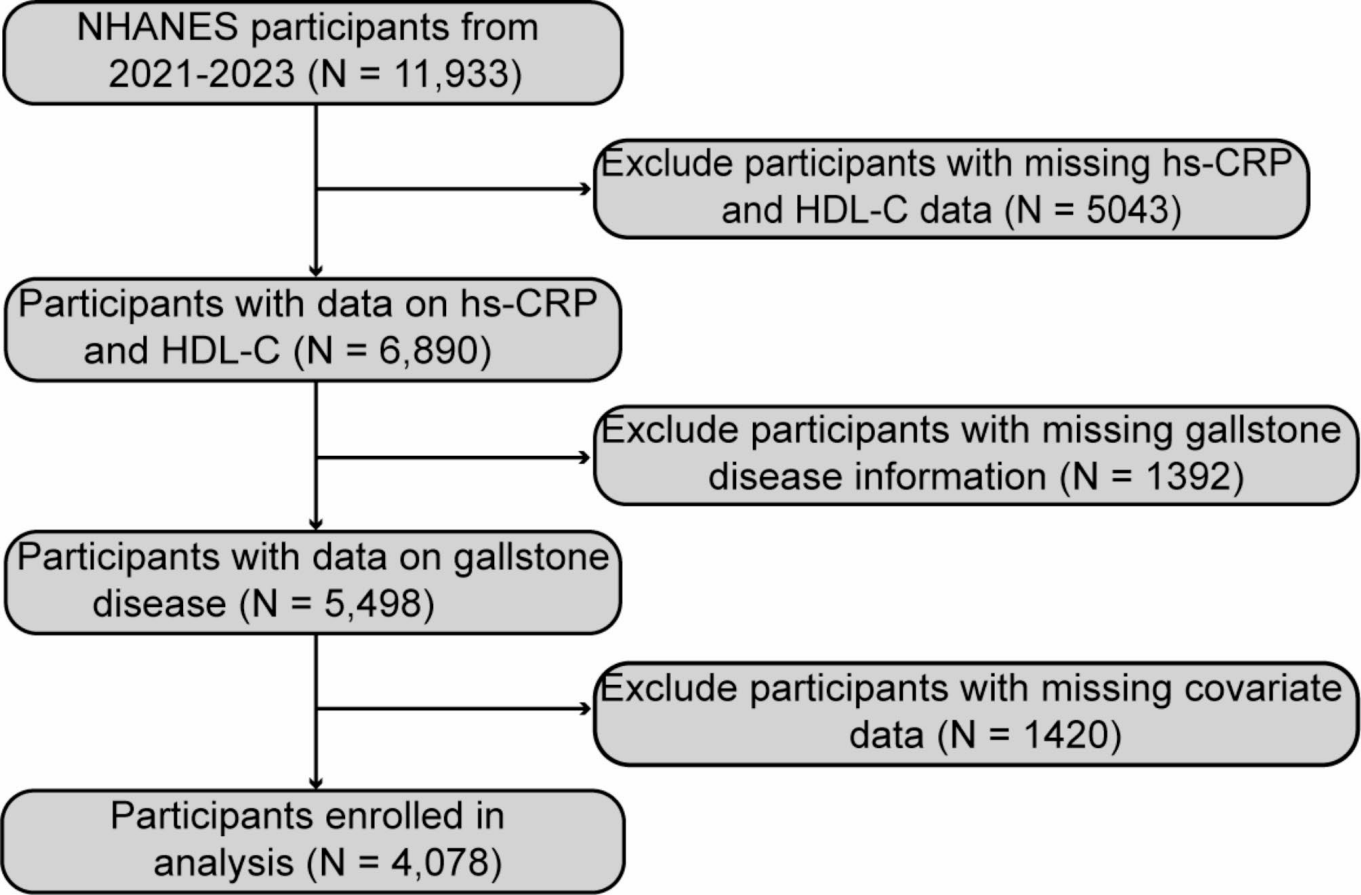
2021–2023 NHANES Study Population Flowchart

At the beginning of the study, 11,933 individuals were enrolled. The exclusion standards were established as follows: (1) individuals with missing hs-CRP and HDL-C data (*N* = 5,043); (2) individuals who did not provide information on gallstone disease (*N* = 1,392); and (3) individuals with missing data on relevant covariates (*N* = 1,420). Ultimately, 4,078 participants were enrolled in the subsequent analysis (Fig. 1).

### Definition and assessment of CHR and gallstones

In this study, the CHR was the exposure variable, which was calculated as the hs-CRP level (mg/L) divided by the HDL-C level (mg/dL). The concentrations of hs-CRP and HDL-C were measured using a two-reagent immunoturbidimetric system and an enzymatic method, respectively. Fasting venous blood was drawn from all participants and analyzed using a Roche Cobas 8000 analyzer. Individuals were separated into four subgroups based on CHR quartiles for further analyses.

The outcome variable, gallstones, was diagnosed based on self-reported answers to the NHANES question (MCQ550) on gallstones: “Has a doctor or other health professional ever told {you/survey participant (SP)} that {you/s/he} had gallstones?”

### Covariates

The demographic data collected in this study included age, sex, marital status, race, and education level, while examination and laboratory data included body mass index (BMI); total cholesterol level; red and white blood cell counts; and platelet, fasting plasma glucose, hemoglobin, and glycohemoglobin levels. The education levels were divided into “less than high school”, high school, and “beyond high school”. The BMI was categorized as underweight (< 18.5 kg/m^2^), normal (18.5 to < 25 kg/m^2^), overweight (25 to < 30 kg/m^2^), or obese (≥30 kg/m^2^) groups [21]. In addition, individuals were asked, “{Have you/Has SP} smoked at least 100 cigarettes in {your/his/her} entire life?” and “{Do you/Does SP} now smoke cigarettes?” and the answers were categorized as never, former, and current smoking [22]. The frequency of alcohol consumption question, “During the past 12 months, about how often did you drink any type of alcoholic beverage?” was used to categorize people as “never consuming alcohol”, “consuming alcohol less than once a month”, or “consuming alcohol at least once a month”. Participants were asked to answer “yes/no” to the following questions: “Has a doctor or other health professional ever told {you/SP} that {you have/s/he/SP has} asthma?”, “Has a doctor or other health professional ever told {you/SP} that {you/s/he} had coronary heart disease?”, and “{Have you/Has SP} ever been told by a doctor or other health professional that {you/s/he} had cancer?” [23]. Diagnosis of hypertension was based on the presence of one of the following conditions: 1) mean systolic blood pressure ≥ 140 mmHg or mean diastolic blood pressure ≥ 90 mmHg on oscillometry and 2) an affirmative answer to “{Have you/Has SP} ever been told by a doctor or other health professional that {you/s/he} had hypertension?”. Diabetes was diagnosed based on the presence of one of the following conditions: 1) an affirmative answer to the question “Other than during pregnancy, {have you/has SP} ever been told by a doctor or health professional that {you have/{he/she/SP} has} diabetes?”; 2) blood glucose in fasting status ≥ 126 mg/dL; 3) taking insulin or diabetic medications; and 4) glycohemoglobin level ≥ 6.5%. MAFLD was diagnosed based on a threshold-controlled attenuation parameter score of 285 dB/m on liver ultrasound transient elastography [24]. This threshold has demonstrated 80% sensitivity and 77% specificity for detecting ≥ 5% hepatic fat content in the United States population [25].

### Data analysis

The complex sampling methodology of the NHANES necessitated the utilization of specific subsampling weights, and the detailed methodology is available on the NHANES website. Continuous variables for baseline data are shown as mean (standard deviation, SD) and were analyzed using the t-test. Frequencies (percentages) are used to present categorical variables, which were tested using the weighted Pearson’s chi-square test. Data were grouped according to CHR quartiles, and the first group (Q1) served as the baseline. Weighted logistic regression was used to show the link between the CHR and gallstones, and three models were constructed based on odds ratios (OR) and 95% confidence intervals (CI). The initial regression model was unadjusted, and Model 2 was adjusted for age, sex, marital status, race, and education level. The final model was based on Model 2 with additional adjustments for alcohol consumption frequency, smoking status, hypertension, diabetes, cancer, coronary heart disease, asthma, and hemoglobin and total cholesterol levels. A potential linear trend was assessed by including CHR quartiles in the model.

In addition, subgroup analyses and interaction tests were applied for determining the association and differences between the CHR and differences in subgroup populations with gallstones. To further evaluate whether the correlation between the CHR and gallstone disease was non-linear, we employed restricted cubic spline (RCS) curve fitting and utilized the Akaike information criterion to determine the optimal model nodes. Assessing the role of MAFLD in the relationship between the CHR and gallstone disease was utilized by mediation analysis. All of the analyses in the study were performed by R (version 4.1.3). The statistical significance standard for two-tailed *P*-values was set at 0.05.

## Results

### Baseline features of participants

The study enrolled 4,087 participants, of which 432 (10.60%) were diagnosed with gallstones. The mean (SD) age of the participants was 53.44 (16.68) years, with a male representation of 50.89%. Participants who were older; female; widowed, divorced, or separated; obese; former smokers; consumed alcohol less than once a month or never; or had cancer, coronary heart disease, asthma, diabetes, hypertension, or MAFLD had a higher risk of gallstones than others (all *P* < 0.05). Additionally, participants with gallstones tended to have higher hs-CRP levels and white blood cell counts but lower levels of HDL-C, total cholesterol, and hemoglobin. Table 1 summarizes weighted features of participants with and without gallstones.

**Table 1:** Weighted features of participants with and without gallstones

Characteristics	Total (*N* = 4078)	With gallstones (*N* = 3646)	Without gallstones	*P* value (*N* = 432)
**Age (years)**,** mean (SD)**	53.44 (16.68)	52.60 (16.78)	60.55 (13.91)	**< 0.001**
**Sex**,** n (%)**				**< 0.001**
Male	1908 (50.89)	1781 (52.92)	127 (29.52)	
Female	2170 (49.11)	1865 (47.08)	305 (70.48)	
**Marital**,** n (%)**				**< 0.001**
Married/Living with partner	2261 (61.94)	2018 (61.92)	243 (62.15)	
Widowed/Divorced/Separated	990 (18.13)	852 (17.24)	138 (27.49)	
Never married	827 (19.93)	776 (20.84)	51 (10.36)	
**Race**,** n (%)**				0.13
Mexican American	258 (6.78)	231 (6.78)	27 (6.73)	
Other Hispanic	379 (8.82)	339 (8.85)	40 (8.60)	
Non-Hispanic White	2607 (64.66)	2311 (64.29)	296 (68.61)	
Non-Hispanic Black	422 (9.54)	391 (9.90)	31 (5.72)	
Other Races	412 (10.20)	374 (10.18)	38 (10.34)	
**BMI**,** n (%)**				**< 0.001**
Underweight	45 (1.21)	41 (1.25)	4 (0.72)	
Normal	1036 (25.88)	987 (27.22)	49 (11.80)	
Overweight	1341 (32.58)	1219 (33.12)	122 (26.93)	
Obese	1656 (40.33)	1399 (38.41)	257 (60.55)	
**Education level**,** n (%)**				0.077
Less than high school	357 (7.18)	312 (7.00)	45 (9.04)	
High school	786 (23.70)	690 (23.33)	96 (27.56)	
Beyond high school	2935 (69.12)	2644 (69.67)	291 (63.40)	
**Alcohol consumption frequency**,** n (%)**				**< 0.001**
At least once a month	2349 (60.78)	2155 (61.94)	194 (48.50)	
Less than once a month	1058 (25.64)	921 (25.10)	137 (31.42)	
never	671 (13.58)	570 (12.96)	101 (20.08)	
**Smoking status**,** n (%)**				**< 0.001**
Current smoking	593 (14.33)	531 (14.51)	62 (12.50)	
Former smoking	1126 (24.62)	973 (23.84)	153 (32.84)	
Never smoking	2359 (61.05)	2142 (61.65)	217 (54.66)	
**Diabetes**,** n (%)**				**< 0.001**
No	3397 (86.18)	3088 (87.24)	309 (74.98)	
Yes	681 (13.82)	558 (12.76)	123 (25.02)	
**Hypertension**,** n (%)**				**< 0.001**
No	2356 (64.53)	2162 (66.27)	194 (46.22)	
Yes	1722 (35.47)	1484 (33.73)	238 (53.78)	
**Cancer**,** n (%)**				**< 0.001**
No	3464 (88.68)	3135 (89.62)	329 (78.83)	
Yes	614 (11.32)	511 (10.38)	103 (21.17)	
**Coronary heart disease**,** n (%)**				0.001
No	3901 (96.67)	3502 (96.94)	399 (93.83)	
Yes	177 (3.33)	144 (3.06)	33 (6.17)	
**Asthma**,** n (%)**				**< 0.001**
No	3331 (81.66)	3009 (82.44)	322 (73.35)	
Yes	747 (18.34)	637 (17.56)	110 (26.65)	
**MAFLD**,** n (%)**				**< 0.001**
No	2635 (64.88)	2417 (66.40)	218 (48.80)	
Yes	1443 (35.12)	1229 (33.60)	214 (51.20)	
**White blood cell (109/L)**,** mean (SD)**	6.86 (1.97)	6.82 (1.95)	7.19 (2.09)	**< 0.001**
**Red blood cell (1012/L)**,** mean (SD)**	4.67 (0.46)	4.67 (0.46)	4.64 (0.45)	0.115
**Platelet (109/L)**,** mean (SD)**	255.29 (65.53)	255.04 (65.05)	257.43 (69.48)	0.473
**Hemoglobin (g/dL)**,** mean (SD)**	14.00 (1.41)	14.02 (1.42)	13.83 (1.31)	0.01
**Total cholesterol (mg/dL)**,** mean (SD)**	189.97 (41.96)	190.51 (41.91)	185.47 (42.17)	0.018
**HDL-C (mg/dL)**,** mean (SD)**	55.04 (14.93)	55.19 (14.97)	53.70 (14.55)	0.049
**hs-CRP (mg/L)**,** mean (SD)**	3.69 (7.01)	3.57 (7.04)	4.71 (6.68)	0.001
**CHR**,** mean (SD)**	0.08 (0.15)	0.07 (0.15)	0.10 (0.14)	0.003

Continuous variables: mean (standard deviation, SD), Categorical variables: number (percentage), *P*-values < 0.001 are shown in bold.

MAFLD, metabolic-associated fatty liver disease; BMI, body mass index; hs-CRP, high-sensitivity C-reactive protein; HDL-C, high-density lipoprotein cholesterol; CHR, high-sensitivity C-reactive protein-to-high-density lipoprotein cholesterol ratio.

Participants were divided into four subgroups according to the weighted CHR quartiles (Table 2). Significant differences were identified among the participants for all variables except coronary heart disease (*P* < 0.05). Notably, the prevalence of gallstones increased significantly among the participants with higher CHR values (Q1:5.05%, Q2:7.94%, Q3:9.07%, Q4:12.56%; *P* < 0.001). With increasing CHR levels, the prevalence of obesity, current or former smoking, high school education, diabetes, MAFLD and white blood cell counts increased significantly (all *P-value* < 0.001).

**Table 2:** Weighted features of participants based on weighted CHR quartiles

Characteristics	Q1 (< 0.01) (*N* = 980)	Q2 (0.01–0.03) (*N* = 1027)	Q3 (0.03–0.08) (*N* = 1042)	Q4 (> 0.08) (*N* = 1029)	*P* valve
**Age (years)**,** mean (SD)**	51.21 (17.79)	55.33 (16.44)	54.79 (16.29)	52.31 (15.88)	**< 0.001**
**Sex**,** n (%)**					
Male	461 (48.91)	533 (55.64)	476 (51.49)	438 (47.53)	**< 0.001**
Female	519 (51.09)	494 (44.36)	566 (48.51)	591 (52.47)	
**Marital**,** n (%)**					**< 0.001**
Married/Living with partner	566 (62.39)	590 (63.45)	567 (61.80)	538 (60.15)	
Widowed/Divorced/Separated	186 (13.85)	275 (20.35)	268 (19.04)	261 (19.26)	
Never married	228 (23.76)	162 (16.20)	207 (19.16)	230 (20.59)	
**Race**,** n (%)**					**< 0.001**
Mexican American	44 (4.47)	57 (6.37)	68 (6.36)	89 (9.91)	
Other Hispanic	67 (6.24)	97 (9.45)	116 (11.11)	99 (8.51)	
Non-Hispanic White	667 (69.76)	678 (64.84)	652 (62.41)	610 (61.64)	
Non-Hispanic Black	89 (8.89)	85 (8.24)	108 (9.92)	140 (11.09)	
Other Races	113 (10.64)	110 (11.10)	98 (10.20)	91 (8.85)	
**BMI**,** n (%)**					**< 0.001**
Underweight	29 (2.90)	9 (0.82)	5 (0.89)	2 (0.22)	
Normal	495 (52.13)	294 (27.44)	160 (14.06)	87 (9.93)	
Overweight	328 (32.58)	429 (41.65)	360 (35.54)	224 (20.56)	
Obese	128 (12.39)	295 (30.09)	517 (49.51)	716 (69.29)	
**Education level**,** n (%)**					**< 0.001**
Less than high school	52 (3.91)	72 (5.34)	110 (9.75)	123 (9.72)	
High school	160 (19.82)	178 (21.95)	217 (24.79)	231 (28.22)	
Beyond high school	768 (76.27)	777 (72.71)	715 (65.46)	675 (62.06)	
**Alcohol consumption frequency**,** n (%)**					**< 0.001**
At least once a month	657 (70.04)	586 (56.11)	572 (59.50)	534 (57.48)	
Less than once a month	188 (18.53)	277 (29.60)	278 (24.83)	315 (29.61)	
never	135 (11.43)	164 (14.29)	192 (15.67)	180 (12.91)	
**Smoking status**,** n (%)**					**< 0.001**
Current smoking	95 (8.60)	136 (14.96)	167 (15.92)	195 (17.85)	
Former smoking	260 (22.20)	286 (24.53)	295 (24.92)	285 (26.82)	
Never smoking	625 (69.20)	605 (60.51)	580 (59.16)	549 (55.33)	
**Diabetes**,** n (%)**					**< 0.001**
No	893 (93.68)	881 (87.85)	867 (86.85)	756 (76.36)	
Yes	87 (6.32)	146 (12.15)	175 (13.15)	273 (23.64)	
**Hypertension**,** n (%)**					**< 0.001**
No	673 (73.52)	601 (66.38)	547 (59.08)	535 (59.16)	
Yes	307 (26.48)	426 (33.62)	495 (40.92)	494 (40.84)	
**Cancer**,** n (%)**					0.027
No	853 (90.11)	846 (86.68)	883 (88.93)	882 (89.02)	
Yes	127 (9.89)	181 (13.32)	159 (11.07)	147 (10.98)	
**Coronary heart disease**,** n (%)**					0.354
No	944 (97.30)	974 (96.07)	1001 (97.11)	982 (96.22)	
Yes	36 (2.70)	53 (3.93)	41 (2.89)	47 (3.78)	
**Asthma**,** n (%)**					0.004
No	824 (83.59)	859 (84.32)	837 (78.98)	811 (79.73)	
Yes	156 (16.41)	168 (15.68)	205 (21.02)	218 (20.27)	
**MAFLD**,** n (%)**					**< 0.001**
No	854 (87.15)	731 (70.67)	593 (57.30)	457 (44.42)	
Yes	126 (12.85)	296 (29.33)	449 (42.70)	572 (55.58)	
**Gallstones**,** n (%)**					**< 0.001**
No	914 (94.95)	931 (92.06)	921 (90.93)	880 (87.44)	
Yes	66 (5.05)	96 (7.94)	121 (9.07)	149 (12.56)	
**White blood cel(109/L)**,** mean (SD)**	6.05 (1.65)	6.52 (1.70)	7.02 (1.85)	7.79 (2.19)	**< 0.001**
**Red blood cell (1012/L)**,** mean (SD)**	4.58 (0.44)	4.68 (0.46)	4.71 (0.46)	4.70 (0.47)	**< 0.001**
**Platelet (109/L)**,** mean (SD)**	244.67 (58.08)	243.87 (56.63)	255.99 (62.68)	276.10 (77.23)	**< 0.001**
**Hemoglobin (g/dL)**,** mean (SD)**	13.96 (1.34)	14.18 (1.36)	14.07 (1.41)	13.78 (1.48)	**< 0.001**
**Total cholesterol (mg/dL)**,** mean (SD)**	187.69 (39.80)	190.58 (42.25)	192.86 (42.89)	188.61 (42.60)	0.027
**HDL-C (mg/dL)**,** mean (SD)**	64.24 (15.67)	56.06 (14.30)	52.27 (12.97)	48.05 (11.73)	**< 0.001**
**hs-CRP (mg/L)**,** mean (SD)**	0.51 (0.22)	1.22 (0.43)	2.76 (0.97)	10.12 (11.64)	**< 0.001**

Continuous variables: mean (standard deviation, SD), Categorical variables: number (percentage), *P*-values < 0.001 are shown in bold.

MAFLD, metabolic-associated fatty liver disease; BMI, body mass index; hs-CRP, high-sensitivity C-reactive protein; HDL-C, high-density lipoprotein cholesterol; CHR, high-sensitivity C-reactive protein-to-high-density lipoprotein cholesterol ratio.

### Logistic regression analysis

Multiple logistic regression analysis was used to analyze the correlation between the CHR and the presence of gallstones (Table 3). Both the original model and the partially adjusted Model 2 (comprising age, sex, marital status, race, and education level) demonstrated a stable statistically significant positive relationship between the CHR and gallstones (Model 1, OR: 2.52, 95% CI: 1.61–3.95, *P* = 0.001; Model 2, OR: 3.23, 95% CI: 1.81–5.75, *P* = 0.001). After incorporating hypertension, diabetes, smoking status, alcohol consumption frequency, coronary artery disease, cancer, asthma, and total cholesterol and hemoglobin levels as covariates into Model 2, a significant correlation was maintained between the CHR and the occurrence of gallstones (OR: 2.65, 95% CI: 1.43–4.93, *P* = 0.006). Thus, the risk of gallstones increased by 165% with each unit increase in the CHR. When the exposure variable was replaced using the CHR quartiles, the strong direct association between the CHR and risk of gallstones remained in Model 3 (*P* for trend < 0.001). Individuals in the CHR Q4 group were twice as likely to have gallstones as those in the reference group (OR: 2.39, 95% CI: 1.68–3.42, *P* < 0.001). In addition, the results revealed a positive and statistically significant relationship between MAFLD and gallstone disease (*P* < 0.001). In Model 3, participants with MAFLD had a higher susceptibility to gallstones (OR: 1.80, 95% CI: 1.40–2.31, *P* < 0.001).

**Table 3:** Multivariable-adjusted associations of CHR and MAFLD with gallstone risk

Exposure		Model 1 OR (95% CI)	*P*-value	Model 2 OR (95% CI)	*P*-value	Model 3 OR (95% CI)	*P*-value
CHR (continuous)		2.52 (1.61–3.95)	0.001	3.233 (1.81–5.75)	0.001	2.65 (1.43–4.93)	0.006
CHR (quartile)							
Quartile 1		Reference		Reference		Reference	
Quartile 2		1.62 (1.12–2.35)	0.026	1.48 (1.02–2.15)	0.057	1.39 (0.956–2.02)	0.105
Quartile 3		1.88 (1.43–2.46)	**< 0.001**	1.66 (1.21–2.29)	0.007	1.49 (1.05–2.12)	0.041
Quartile 4		2.70 (1.98–3.69)	**< 0.001**	2.70 (1.93–3.78)	**< 0.001**	2.39 (1.68–3.42)	**< 0.001**
*P* for trend			**< 0.001**		**< 0.001**		**< 0.001**
MAFLD							
No		Reference		Reference		Reference	
Yes		2.07 (1.71–2.51)	**< 0.001**	2.05 (1.69–2.49)	**< 0.001**	1.80 (1.40–2.31)	**< 0.001**

Model 1: unadjusted; Model 2: adjusted for age, sex, race, marital status, and education level; Model 3: Model 2 adjusted for alcohol consumption frequency, smoking status, hypertension, diabetes, cancer, coronary heart disease, asthma, and hemoglobin and total cholesterol levels. *P*-values < 0.001 are shown in bold

MAFLD, metabolic-associated fatty liver disease; OR, odds ratio; CI, confidence interval; CHR, high-density C-reactive protein-to-high-density lipoprotein cholesterol ratio

The positive correlation between the CHR quartiles and MAFLD was stable and significant (all *P* for trend < 0.001) (Table 4). In Models 1 and 2, the risk of MAFLD in the Q4 group was approximately eight times higher than that in the reference (Q1) group (Model 1 Q4, OR: 8.49, 95% CI: 5.73–12.56, *P* < 0.001; Model 2 Q4, OR: 8.66, 95% CI: 5.86–12.79, *P* < 0.001). According to Model 3, additionally adjusting for hypertension, diabetes, smoking status, alcohol consumption frequency, coronary heart disease, cancer, asthma, and total cholesterol and hemoglobin levels, the Q4 group was still likely to develop MAFLD (OR: 7.42, 95% CI: 5.03–10.92, *P* < 0.001).

**Table 4:** CHR and MAFLD risk across multivariable-adjusted models

Exposure	Model 1 OR (95% CI)	*P*-value	Model 2 OR (95% CI)	*P*-value	Model 3 OR (95% CI)	*P*-value
CHR (continuous)	46.37 (12.99–165.51)	**< 0.001**	59.66 (17.41–204.50)	**< 0.001**	32.51 (9.63–109.71)	**< 0.001**
CHR (quartile)	Reference		Reference		Reference	
Q1						
Q2	2.81 (2.13–3.73)	**< 0.001**	2.60 (1.96–3.45)	**< 0.001**	2.36 (1.76–3.17)	**< 0.001**
Q3	5.05 (3.46–7.39)	**< 0.001**	4.83 (3.27–7.14)	**< 0.001**	4.35 (2.94–6.43)	**< 0.001**
Q4	8.49 (5.73–12.56)	**< 0.001**	8.66 (5.86–12.79)	**< 0.001**	7.42 (5.03–10.92)	**< 0.001**
*P* for trend		**< 0.001**		**< 0.001**		**< 0.001**

Model 1: unadjusted; Model 2: adjusted for age, sex, race, marital status, and education level; Model 3: Model 2 adjusted for alcohol consumption frequency, smoking status, hypertension, diabetes, cancer, coronary heart disease, asthma, and hemoglobin and total cholesterol levels. *P* values < 0.001 are shown in bold

MAFLD, metabolic-associated fatty liver disease; OR, odds ratio; CI, confidence interval; CHR, high-density C-reactive protein-to-high-density lipoprotein cholesterol ratio

### Nonlinear relationship between gallstones and the CHR

**Fig. 2:**
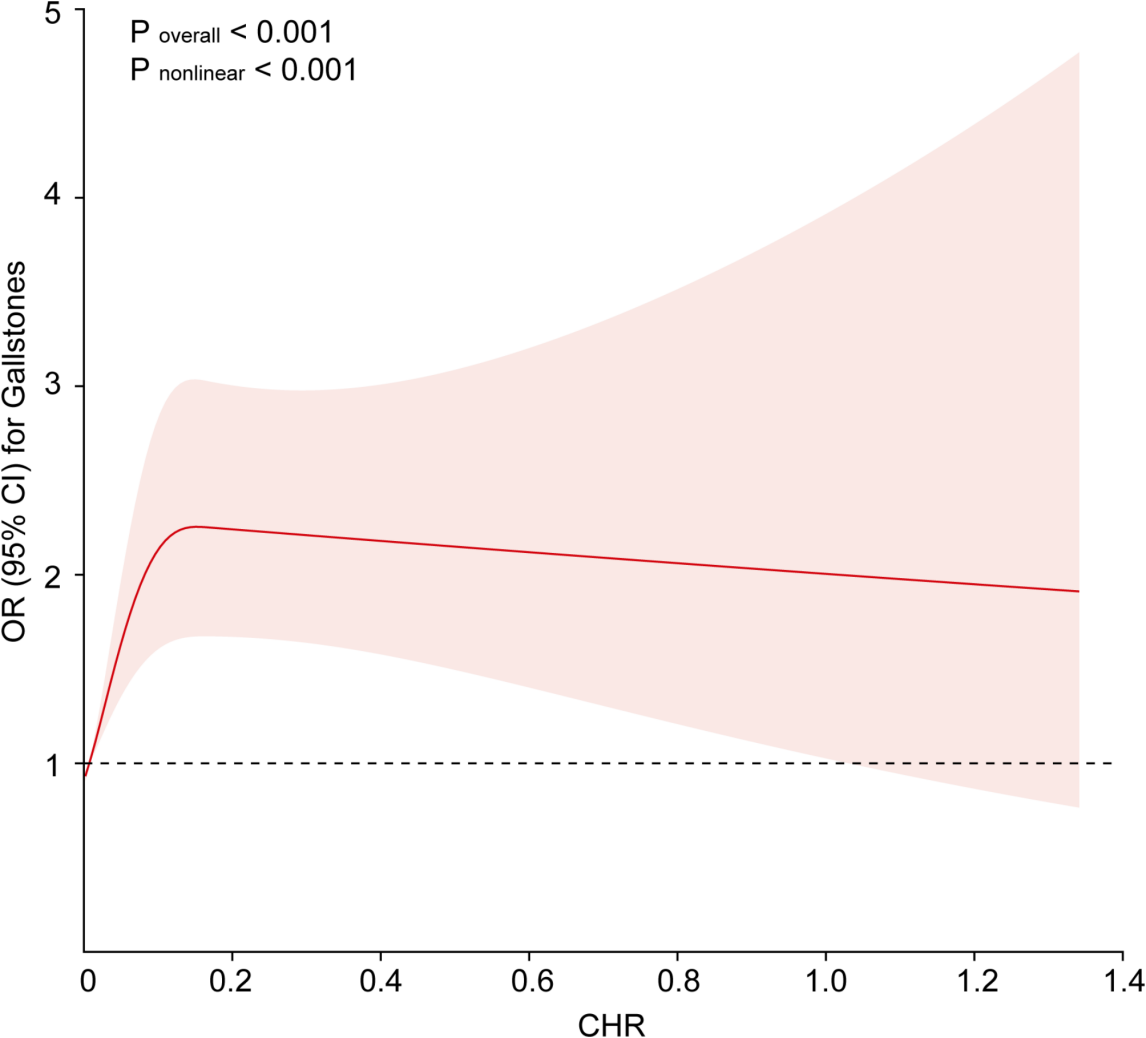
RCS analysis of the CHR and gallstones

The RCS curve for continuous CHR and risk of gallstones is shown in Fig. 2. After adjusting for all possible confounding variables (age, sex, marital status, race, education level, alcohol consumption frequency, smoking status, hypertension, diabetes, cancer, coronary heart disease, asthma, and hemoglobin and total cholesterol levels), the CHR had a nonlinear association with the risk of gallstones (*P*_overall_ < 0.001, *P*_nonlinear_ < 0.001).

### Subgroup analyses

**Fig. 3:**
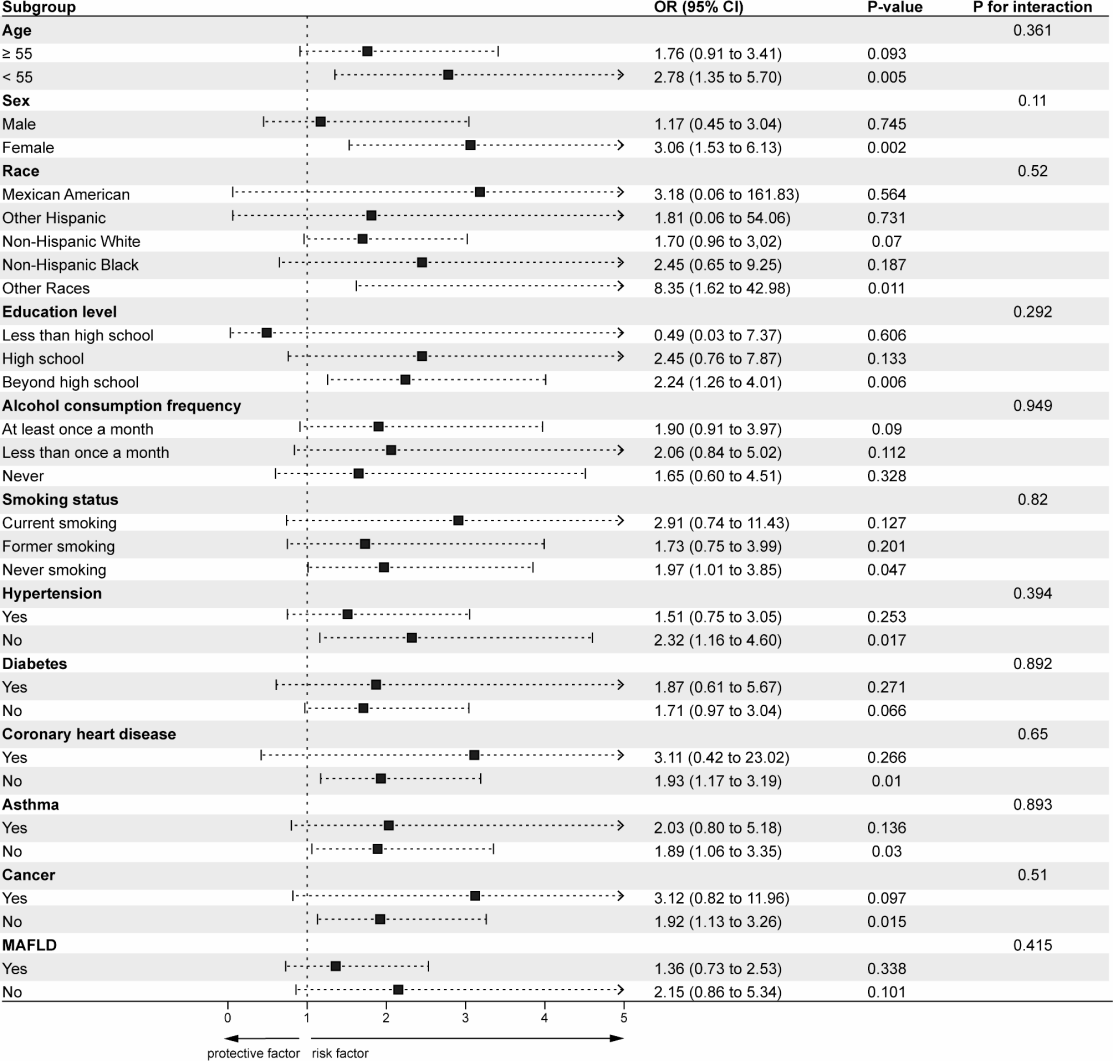
Subgroup analyses of the association between the CHR and risk of gallstones

Subgroup analyses were conducted to further investigate the robustness and possible differences in the association between the CHR and the risk of gallstone disease (Fig. 3). No significant interactions were observed between the CHR and age (*P* = 0.361), sex (*P* = 0.11), race (*P* = 0.52), education level (*P* = 0.292), alcohol consumption frequency (*P* = 0.949), smoking status (*P* = 0.82), hypertension (*P* = 0.394), diabetes (*P* = 0.892), coronary heart disease (*P* = 0.65), asthma (*P* = 0.893), cancer (*P* = 0.51), and MAFLD (*P* = 0.415). The relationship between the CHR and an increased risk of gallstone disease was significant in the following subgroups: younger than 55 years, female, other races, education beyond high school, without hypertension, without coronary heart disease, without asthma, without cancer, and never smoked (all *P* < 0.05).

### Mediation analysis

**Fig. 4:**
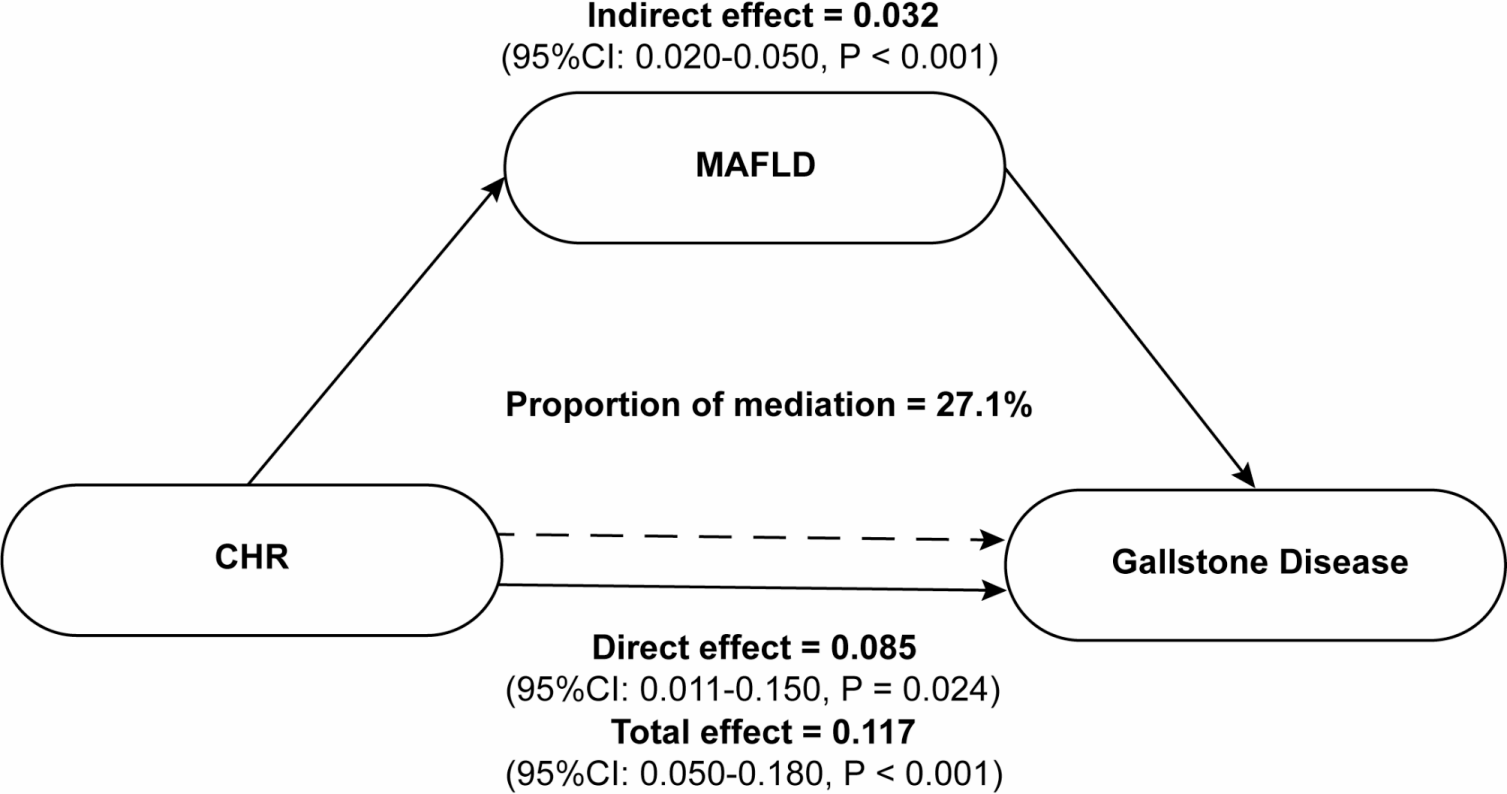
Mediating effect of MAFLD on the association between the CHR and gallstone prevalence

The role of MAFLD in this association was evaluated using mediation analysis. After accounting for confounding factors, CHR was found to have direct and indirect effects on gallstones, and approximately 27.1% of the indirect effect was explained by MAFLD (*P* < 0.001) (Fig. 4).

## Discussion

This study investigated the mediating effect of MAFLD on the association between the CHR and gallstones using the NHANES data from August 2021 to August 2023. Ultimately, 4,078 participants were enrolled in the study. Baseline statistical and logistic regression analyses showed a significant positive association between the CHR and gallstones. Even with full adjustments for confounding covariates, the risk of gallstone disease increased by 165% with each unit increase in the CHR. The RCS curve confirmed that the relationship was nonlinear. Subgroup analysis found that the association was more pronounced in patients younger than 55 years of age, females, from other races, and in those with an educational level beyond high school, without hypertension, without coronary heart disease, without asthma, without cancer, and those who had never smoked. The interaction tests found no significant differences between the subgroups. Finally, MAFLD had a partial mediating effect on the association between the CHR and gallstone disease, suggesting that clinicians should emphasize MAFLD in the early prevention and management of patients with gallstones, while carrying out anti-inflammatory and lipid management strategies.

As a new biological marker, the CHR consists of the hs-CRP level, which represents inflammatory responses, and the HDL-C, which is involved in lipid metabolism. This study is the first to identify the CHR-MAFLD-gallstone pathway, which can be explained by several potential biological mechanisms. The development of MAFLD in individuals with high CHR may be mediated through two key pathophysiological mechanisms: on the one hand, inflammation of the adipose tissue (e.g., the release of the proinflammatory cytokine tumor necrosis factor) promotes insulin resistance, thereby releasing large amounts of free fatty acids and causing hepatic steatosis [26]. On the other hand, HDL-C not only facilitates reverse cholesterol transport but also exerts anti-inflammatory effects by suppressing the expression of vascular cell adhesion molecule 1 expression and preventing the adhesion of lymphocytes or monocytes to the vascular endothelium [27]. Notably, the possibility of gallstones increased by 80% in participants with MAFLD compared to that in normal participants in Model 3. A meta-analysis by Gu et al. also identified this significant positive correlation and showed that the gallstones incidence was higher in females (OR: 4.18, CI: 1.21–14.37) and in participants with a high BMI (OR: 1.80, CI: 1.36–2.56) [28]. Mediation analysis further identified MAFLD as a partial mediator, accounting for 27.1% of the total effect in the CHR-gallstone association. This may be due to the increased volume of lipid-laden hepatocytes in MAFLD, where hepatic sinusoidal blood flow and microperfusion are reduced, and hypoxia-inducible factor 1α inhibits the expression of aquaporin, which promotes bile concentration and gallstone formation [29–32]. It has also been suggested that MAFLD can impair cholesterol clearance and disrupt bile acid homeostasis by inducing hepatic inflammation, fibrotic processes, and dysregulation of gut-hepatic interactions, which collectively drive bile supersaturation and gallstone formation [33–35]. Furthermore, the included covariates (e.g., diabetes, coronary heart disease, and hypertension) may influence MAFLD and gallstone pathogenesis by modulating lipid metabolism and inflammatory responses [24, 32]. These confounders may also exhibit synergistic or antagonistic interactions with CHR, thus necessitating further mechanistic studies to delineate their complex interplay in disease progression.

The findings of this study provide a new idea for the prevention and hierarchical management of gallstones. First, CHR, as a composite marker of inflammation and metabolism, can be included in the screening system of high-risk groups. For individuals with elevated CHR, anti-inflammatory therapy and lifestyle interventions (e.g., low-fat diet, exercise) are recommended to reduce the risk of gallstones by lowering hs-CRP and increasing HDL-C. Secondly, MAFLD can be used as an intermediating factor for intervention, suggesting that the management of hepatic steatosis may indirectly block the gallstone formation pathway. However, future clinical trials need to validate the long-term effects of these strategies.

This study has several advantages over previous studies. First, the NHANES is a nationally representative survey on American adults utilizing complex multistage probability sampling to ensure representativeness and accuracy. Second, confounding covariates were meticulously selected to mitigate the potential impact of bias on the results, thereby enhancing the credibility and generalizability of the findings. Third, subgroup and mediation analyses were performed to further investigate the stability of the link within the CHR and gallstones in different subgroup populations as well as the mediating effect of MAFLD on the relationship. However, this study had some limitations. First, the diagnosis of gallstones in the NHANES data relied on self-answered questionnaires, which may be influenced by recall bias and risk of misclassification. Second, although we adjusted for multiple covariates, potential confounding factors such as dietary habits and physical activity levels were not incorporated into the analytical models, which may introduce residual confounding bias. Third, a causal relationship between the CHR and gallstones could not be concluded because of the cross-sectional nature of the study. Finally, the mediation analysis revealed only a partial mediating effect, suggesting the potential involvement of additional pathways (e.g., gut microbiota-related mechanisms) that warrant further validation.

## Conclusion

This study suggests a significantly positive association between increased CHR values and an increased risk of gallstones, with possible partial mediation by MAFLD. This implies that clinicians should manage the CHR within normal limits and prevent the development of MAFLD to decrease the incidence of gallstones and alleviate the burden on the healthcare system. However, further thorough investigations are required to clarify the underlying mechanisms.

## Data Availability

This study analyzed public NHANES datasets. These datasets are available at https://www.cdc.gov/nchs/nhanes/index.htm.

## Abbreviations

BMI: Body mass index
CHR: High-sensitivity C-reactive protein-to-high-density lipoprotein cholesterol ratio
CI: Confidence interval
HDL-C: High-density lipoprotein cholesterol
hs-CRP: High-sensitivity C-reactive protein
MAFLD: Metabolic associated fatty liver disease
NCHS: National Center for Health Statistics
NHANES: National Health and Nutrition Examination Survey
OR: Odds ratio
RCS: Restricted cubic spline
SD: Standard deviation
SP: Survey participant
